# Effects of Dietary Oat Beta-Glucans on Colon Apoptosis and Autophagy through TLRs and Dectin-1 Signaling Pathways—Crohn’s Disease Model Study

**DOI:** 10.3390/nu13020321

**Published:** 2021-01-22

**Authors:** Łukasz Kopiasz, Katarzyna Dziendzikowska, Małgorzata Gajewska, Michał Oczkowski, Kinga Majchrzak-Kuligowska, Tomasz Królikowski, Joanna Gromadzka-Ostrowska

**Affiliations:** 1 Department of Dietetics, Institute of Human Nutrition Sciences, Warsaw University of Life Sciences, Nowoursynowska 159c, 02-776 Warsaw, Poland; lukasz_kopiasz@sggw.edu.pl (Ł.K.); michal_oczkowski@sggw.edu.pl (M.O.); tomasz_krolikowski@sggw.edu.pl (T.K.); joanna_gromadzka_ostrowska@sggw.edu.pl (J.G.-O.); 2 Department of Physiological Sciences, Institute of Veterinary Medicine, Warsaw University of Life Sciences, Nowoursynowska 159, 02-776 Warsaw, Poland; malgorzata_gajewska@sggw.edu.pl (M.G.); kinga_majchrzak@sggw.edu.pl (K.M.-K.)

**Keywords:** oat beta-glucan, *colitis*, Crohn’s disease, apoptosis, autophagy, TLRs, Dectin-1, rats

## Abstract

Background: Crohn’s disease (CD) is characterized by chronic inflammation of the gastrointestinal tract with alternating periods of exacerbation and remission. The aim of this study was to determine the time-dependent effects of dietary oat beta-glucans on colon apoptosis and autophagy in the CD rat model. Methods: A total of 150 Sprague–Dawley rats were divided into two main groups: healthy control (H) and a TNBS (2,4,6-trinitrobenzosulfonic acid)-induced *colitis* (C) group, both including subgroups fed with feed without beta-glucans (βG−) or feed supplemented with low- (βGl) or high-molar-mass oat beta-glucans (βGh) for 3, 7, or 21 days. The expression of autophagy (LC3B) and apoptosis (Caspase-3) markers, as well as Toll-like (TLRs) and Dectin-1 receptors, in the colon epithelial cells, was determined using immunohistochemistry and Western blot. Results: The results showed that in rats with *colitis*, after 3 days of induction of inflammation, the expression of Caspase-3 and LC3B in intestinal epithelial cells did not change, while that of TLR 4 and Dectin-1 decreased. Beta-glucan supplementation caused an increase in the expression of TLR 5 and Dectin-1 with no changes in the expression of Caspase-3 and LC3B. After 7 days, a high expression of Caspase-3 was observed in the colitis-induced animals without any changes in the expression of LC3B and TLRs, and simultaneously, a decrease in Dectin-1 expression was observed. The consumption of feed with βGl or βGh resulted in a decrease in Caspase-3 expression and an increase in TLR 5 expression in the CβGl group, with no change in the expression of LC3B and TLR 4. After 21 days, the expression of Caspase-3 and TLRs was not changed by colitis, while that of LC3B and Dectin-1 was decreased. Feed supplementation with βGh resulted in an increase in the expression of both Caspase-3 and LC3B, while the consumption of feed with βGh and βGl increased Dectin-1 expression. However, regardless of the type of nutritional intervention, the expression of TLRs did not change after 21 days. Conclusions: Dietary intake of βGl and βGh significantly reduced colitis by time-dependent modification of autophagy and apoptosis, with βGI exhibiting a stronger effect on apoptosis and βGh on autophagy. The mechanism of this action may be based on the activation of TLRs and Dectin-1 receptor and depends on the period of exacerbation or remission of CD.

## 1. Introduction

Inflammatory bowel disease (IBD) is becoming an increasingly common disease in the population of developed countries. One of the reasons for the increase in its incidence is changes in lifestyle, including eating habits, which have been observed over the past few decades. The transition from a diet based on natural or mildly processed products to a Western-type diet is a consequence of change from a rural lifestyle to an urban one, which took place on a large scale in the 20th century [1]. IBD is a disease characterized by chronic inflammation of the gastrointestinal tract, and includes ulcerative colitis (UC) and Crohn’s disease (CD) [2]. The etiology of both these diseases is multifactorial, in which interactions between genetic and environmental factors as well as changes in the profile of the gut microbiota (dysbiosis) consequently contribute to disturbances in immune response mechanisms, leading to the development of inflammation [3]. UC first appears in the mucosa and submucosa of the large intestine, while in CD the chronic inflammatory process may occur in any part of the gastrointestinal tract, covering the entire thickness of its wall [4,5].

One of the main effects of chronic inflammation related to CD is damage to the intestinal epithelium and a significant increase in the programmed death of intestinal epithelial cells (IECs). In addition, disturbances occur in the mechanisms regulating the metabolic activity of cells, including autophagy [6]. Apoptosis contributes to the dynamic control of cells by removing damaged cells or that function abnormally without triggering inflammatory response or oxidative stress, thus ensuring appropriate tissue function. Simultaneously, autophagy plays an important role in maintaining the homeostasis of living organisms, as they regulate the basic metabolic functions and pathogenesis of many diseases, including malignant neoplasms, neurodegenerative and cardiovascular diseases, and IBD [6,7,8]. Both these processes regulate tissue homeostasis and response to extracellular factors, including the production of proinflammatory cytokines, and intensification of the apoptosis of IECs has been reported in people with IBD and in in vivo studies on animal models [9]. Caspase-3 is considered to be one of the key executive enzymes of apoptosis, the activation of which is common to both extrinsic and intrinsic pathways, as well as is dependent on granzyme B [10]. A protein specific to autophagy is LC3, which is involved in the final formation of the autophagosome [8]. In mammalian cells, this protein occurs in three isoforms—LC3A, LC3B, and LC3C—of which the LC3B form is the marker of autophagosomal activity [11,12].

In the light of the latest research, factors regulating the proliferation, autophagy, and apoptosis of intestinal cells include membrane Toll-like receptors (TLRs), such as TLR 4, TLR 5, and TLR 9, and Dectin-1 receptor. Activation of TLRs and Dectin-1 receptors stimulates the immune response. The main function of these receptors is the recognition of bacterial and fungal antigens. They also recognize a wide variety of bacterial and fungal ligands, of which the best known are lipopolysaccharide (LPS), flagellin, and triacyl lipopeptides. Plant-origin polysaccharides, including beta-glucans, are ligands that bind to TLRs and Dectin-1 receptors. Due to their specific structure, beta-glucans exhibit a number of health-promoting properties, and their biological activity results from their ability to bind with TLRs and Dectin-1 receptors, as well as with complement receptor 3 and type C lectin [13,14]. Beta-glucans are recognized by different cells expressing TLRs and Dectin-1 receptors, which in turn activate them [15,16,17]. It should be emphasized that the activation of Dectin-1 receptor by oat beta-glucans is influenced by the size of these polysaccharide molecules and is greater when they are subjected to preliminary digestion, i.e., when large molecules are broke down into smaller ones [17]. Dectin-1 receptors are involved, among others, in regulating the differentiation of macrophages and dendritic cells during ongoing inflammation, as well as modulating the immune response and controlling the recruitment of autophagy-related proteins, mainly LC3 [18,19,20,21]. The deficiency of TLRs inhibits the proliferation and differentiation of IECs and increases apoptosis, which affects the reconstruction of the intestinal epithelium [22,23]. This is very important with respect to the treatment of IBD, because due to chronic inflammation, the IECs are vulnerable to damage caused by high levels of reactive oxygen species (ROS). Moreover, as a consequence of the increased secretion of proinflammatory cytokines by immune cells, the permeability of the intestinal barrier increases [24,25].

The results of our research have shown the beneficial anti-inflammatory and indirect antioxidant effects of oat beta-glucans with low and high molar mass. These properties have been demonstrated in both the LPS-induced enteritis model [26,27] and the TNBS (2,4,6-trinitrobenzosulfonic acid)-induced colitis model [28,29], but the mechanisms underlying these effects have not yet been elucidated. Therefore, this study aimed to determine the effect of two oat beta-glucan forms with different molar mass on the activity of autophagy and apoptosis in the colonocytes of rats with colitis. Rectal administration of an ethanolic solution of TNBS causes strictly localized transmural inflammatory lesions in the large intestine, with dense lymphocyte infiltration and secretion of proinflammatory cytokines in the whole wall, which are characteristic of CD in humans. Moreover, this animal model is characterized by reproducibility, technical simplicity and low cost [30,31]. The molecular effects of both beta-glucan forms have been analyzed at different stages of colitis development.

## 2. Materials and Methods

### 2.1. Animals and Experimental Design

**Figure 1:**
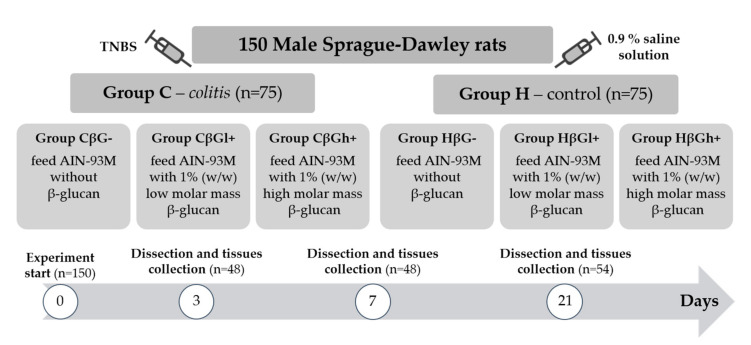
Scheme of experimental design of the study. TNBS: 2,4,6,-trinitrobenzenesulfonic acid alcohol solution.

The experiment was performed on 150 adult male Sprague–Dawley rats (Charles River Laboratories, Sulzfeld, Germany) with an initial body weight of 414.8 ± 1.3 g. The rats were divided into two main groups—an experimental group (*colitis* group—C) with colitis induced by one-time rectal administration of 1 mL of 2,4,6-trinitrobenzenesulfonic acid (Sigma Aldrich, Darmstadt, Germany) solution (150 mg/kg bw) dissolved in 50% ethanol and a control group (H) without colitis, which was given 0.9% NaCl in the same way. Administration of TNBS was performed using the polyethylene catheter (FTP-18-75-50; Instech Laboratories, Inc., Plymouth Meeting, PA, USA) according to Parra et al. (2015) [32]. The animals from both groups were then divided into three nutritional subgroups, which received a diet with different types of supplementation for 3, 7, or 21 days—AIN-93M feed with 1% (*w*/*w*) low-molar-mass oat beta-glucans (βGl+ group), AIN-93M feed with 1% (*w*/*w*) high-molar-mass oat beta-glucans (βGh+ group), and AIN-93M feed without beta-glucans (βG− group) (Figure 1). A detailed description of the in vivo experiment and the method used for the extraction of beta-glucans from oats as well as the composition of the three types of rat feed used in this study is provided in our previous papers [28,29].

The large intestine samples obtained for histological and immunohistochemical analysis were fixed in 10% buffered formaldehyde and routinely embedded in paraffin. Then, 5-µm sections were cut from each paraffin block and fixed on microscope glass slides. The slides prepared were used for further laboratory analyses.

The animal experiment was conducted after the approval of the II Local Animal Care and Use Committee in Warsaw (Resolution # 60/2015). All the procedures will be designed and conducted according to Polish and EU law regulations and with respect to 3R rules (Replacement, Reduction and Refinement).

### 2.2. Western Blot Analysis

Colon tissue samples were homogenized in RIPA buffer (50 mM Tris, pH 7.5, 150 mM NaCl, 1 mM EDTA, 1% NP-40, 0.25% Na-deoxycholate, and 1 mM PMSF) supplemented with protease inhibitor cocktail and phosphatase inhibitor cocktail (Sigma-Aldrich) with a tissue homogenizer (Bio-Gen PRO 200; PRO Scientific, Oxford, CT, USA). Following the mechanical disruption of tissue, the homogenates were incubated for 30 min at 4 °C. The lysates were cleared for 30 min at 14,000 rpm, and supernatants were collected. Protein concentration in the lysates was determined using Thermo Scientific™ Pierce™ BCA Protein Assay Kit according to the producer’s instructions (Thermo Scientific, Waltham, MA, USA). Then, the proteins (50 μg) were resolved by sodium dodecyl sulfate-polyacrylamide gel electrophoresis and transferred onto PVDF membrane (Sigma-Aldrich). The membranes were blocked in 5% (*w*/*v*) nonfat dry milk in TBS (20 mM Tris-HCl, 500 mM NaCl) containing 0.5% Tween20. Next, they were incubated under gentle shaking at 4 °C overnight with primary antibodies, including anti-Cleaved Caspase-3 antibody (Cell Signaling Technology, Danvers, MA, USA), anti-LC3B antibody (Novus Biologicals, Centennial, CO, USA), anti-Beclin-1 antibody (Novus Biologicals, Centennial, CO, USA), and anti-β-actin (8H10D10) mouse monoclonal antibody (#3700; Cell Signaling Technology, Danvers, MA, USA). The membranes were incubated with chosen primary antibodies at a dilution range between 1:200 and 1:1000, depending on the antibody. Following incubation, the membranes were washed three times for 15 min and incubated with appropriate secondary antibodies conjugated with IR fluorophores: IRDye^®^ 680 or IRDye^®^ 800 CW (at 1:5000 dilution). Odyssey Infrared Imaging System (LI-COR Biosciences, Lincoln, NE, USA) was used to analyze the protein expression. Scan resolution of the instrument was set at 169 μm, and the intensity at 4. Quantification of the integrated optical density (IOD) was performed using the analysis software provided with the Odyssey scanner (LI-COR Biosciences). Immunoblot analysis was carried out on samples obtained from three independent experiments. For the purpose of publication, the color immunoblot images were converted into black and white images in the Odyssey software.

### 2.3. Immunohistochemical Staining

Five-micrometer-large intestine sections were deparaffinized in xylene and rehydrated in a series of decreasing concentrations of ethanol. To recover the antigen, the slides were placed in citrate buffer (pH 6.0) and boiled in a microwave two times for 5 min. After cooling and washing with distilled water, the samples were incubated in the Peroxidase Blocking Reagent (Dako, Denmark) for 13 min at room temperature. Then, these samples were incubated at room temperature in 2% bovine serum albumin (BSA) (Sigma Aldrich, Germany) for 30 min. Next, they were treated with the following primary antibodies (diluted in 2% BSA): mouse anti-TLR 4 antibody (1:100 dilution; Novus Biologicals, Centennial, CO, USA), rabbit anti-Dectin-1/CLEC7A antibody (1:400 dilution; Novus Biologicals, Centennial, CO, USA), rabbit anti-TLR 5 antibody (1:500 dilution; Novus Biologicals, Centennial, CO, USA), rabbit anti-TLR 6 antibody (1:250 dilution; Novus Biologicals, Centennial, CO, USA), rabbit anti-LC3B antibody (1:500 dilution; Novus Biologicals, Centennial, CO, USA), rabbit anti-Cleaved Caspase-3 antibody (1:400 dilution; Cell Signaling Technology, Danvers, MA, USA). The slides with antibodies were incubated overnight in a refrigerator at +4 °C.

EnVision kit (Dako, Glostrup Kommune, Denmark) was used for staining the slides. After incubating overnight and washing with phosphate-buffered saline, labeled polymers consisting of secondary antimouse or antirabbit antibodies conjugated with HRP enzyme complex was used (Dako). To obtain brown staining, 3,3′-diaminobenzidine was applied, which was washed with water after 25 s. Then, hematoxylin was used for nuclei counterstaining. Finally, the samples were dehydrated using a series of alcohols of increasing concentration and xylene, and preserved by sticking coverslips.

A negative control (staining without primary antibodies) was used for each immunohistochemical experiment.

### 2.4. Image Analysis

The immunohistochemically stained slides were observed under a ×20 objective lens in a NIKON Eclipse Ti2 microscope. In the recorded photos, 20 areas of colonocytes were marked, and the colorimetric intensity (brown color reflecting antigen expression) and object area were measured by using NIS-Elements BR 5.01 program. In the next step, IOD was calculated from the following formula:

$$\mathrm{Integrated}\;\mathrm{Optical}\;\mathrm{Density}\;\left(\mathrm{IOD}\right)=\frac{Object\;area}{Measured\;area}\;\times \;Mean\;intensity$$

### 2.5. Statistical Analysis

The obtained data were analyzed using Statistica software (version 13.1 PL; StatSoft, Cracow, Poland). The normality of distribution and equality of variance were determined for all data. Statistical analysis was carried out in two stages. In the first stage, for each time point (3, 7, and 21 days), a two-way analysis of variance (ANOVA) (colon inflammation vs. dietary intervention) was performed, while in the second stage, a three-way ANOVA (colon inflammation vs. dietary intervention vs. period of its use) was carried out. The significance of differences between the groups was determined by the Tukey post hoc test. In addition, the results of all nutritional subgroups were compared with the control subgroup (HβG−) by the Dunnett post hoc test. The differences were considered statistically significant at *p* < 0.05. To assess the time-dependent effect between the experimental factors, Fisher’s linear discriminant (FLD) analysis was performed using R statistics software (version 3.1.3; www.r-project.org).

## 3. Results

### 3.1. Histological Changes in the Large Intestine

**Figure 2:**
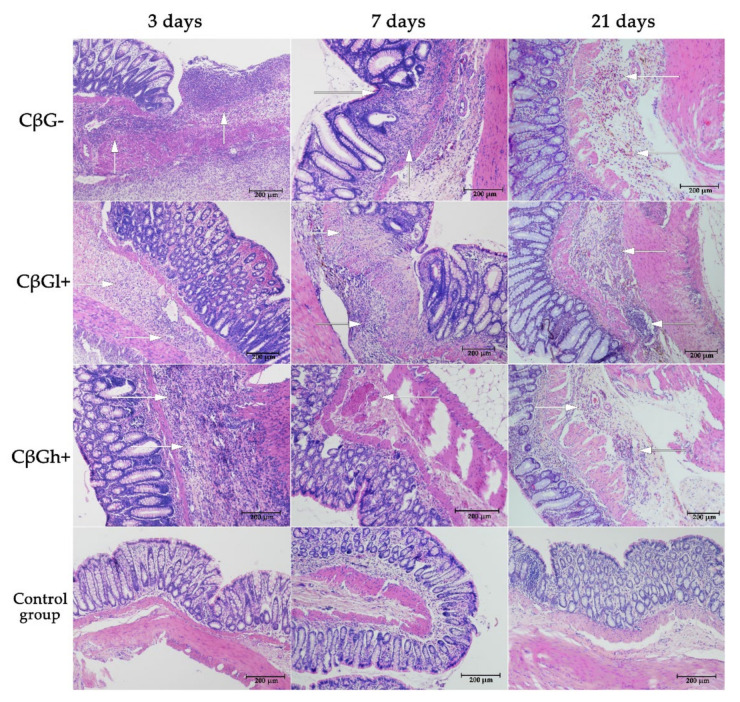
Microscopic changes in the colon caused by TNBS-induced inflammation. White arrows indicate diffused multifocal inflammation (lymphocyte infiltration) of the submucosa of varying severity.

Histological analysis revealed extensive inflammation in the colon tissue of rats in the CβG− group (Figure 2). The lesions were of transwall nature, covering not only mucosa but also the deeper layers of the intestinal wall, which is a characteristic of CD. Inflammation in the colon (colitis) was confirmed in the same animals not only histologically but also biochemically, including analysis of colon level of pro-inflammatory cytokines (i.e., Il-1; Il-6 or TNF alfa) and CRP protein [28]. These microscopic and biochemical observations also confirmed the results of gene expression analysis of pro-inflammatory cytokines in colon tissue affected by colitis, including *Ifng* and *Tnf* after 3 days and *Il21* after 21 days (Figure S9). In the groups of animals fed with feed supplemented with oat beta-glucans during 21 days, these lesions were clearly reduced, as was presented in our previous study [28]. The expression of pro-inflammatory cytokine genes was also significantly reduced (Figure S9). Hematoxylin-eosin staining showed that the histopathology of the colon tissue in the control groups was within the normal limits.

### 3.2. Dectin-1 and Toll-like Receptors 4, 5, and 6 Expression in the Colonocytes

**Figure 3:**
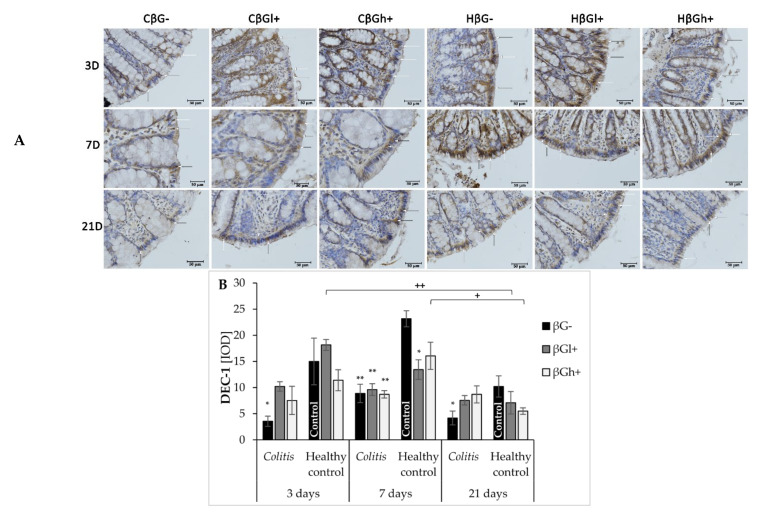
Dectin-1 (DEC-1) expression in colonocytes: results of the immunohistochemical analysis. White arrows indicate colonocytes with high expression of the Dectin-1 (brown precipitate). (**A**)—Light micrographs imaged under the NIKON Eclipse Ti2 microscope (×40 magnification). Dectin-1 antigen is represented by a brown precipitate in the colonocytes. (**B**)—Changes in the expression of DEC-1 in colonocytes presented (mean ± SE) as integrated optical density (IOD). * Significantly different from the control group (healthy control βG−) at the same time point according to the Dunnett post hoc test (* *p* < 0.05, ** *p* < 0.01). ^+^ Significantly different from the same subgroups at another time point according to the Tukey post hoc test (^+^ *p* < 0.05, ^++^ *p* < 0.01).

The expression of Dectin-1 beta-glucan receptor is presented in Figure 3. As shown by ANOVA, the expression of this receptor in colonocytes was lower in the rats with TNBS-induced *colitis* (ANOVA, *p* < 0.001; Figure S1A), whereas dietary intervention with oat beta-glucans resulted in a change in its expression after 3 and 21 days of feeding, which was confirmed by ANOVA. Furthermore, one-way ANOVA showed that the expression of Dectin-1 was lower after 21 days (ANOVA, *p* < 0.001; Figure S1B). After 7 days, induced *colitis* caused a significant decrease in Dectin-1 expression in all dietary subgroups compared with the control group (HβG−) (for each subgroup: Dunnett post hoc test, *p* < 0.01). In addition, analysis of variance showed the effect of interaction between time after TNBS/saline administration and the occurrence of inflammation on Dectin-1 expression (ANOVA, *p* < 0.01; Figure S1C). In the control groups, 3 and 7 days after saline solution administration, the expression of this receptor was significantly higher than in the colitis groups at the same time point and in the control group after 21 days.

**Figure 4:**
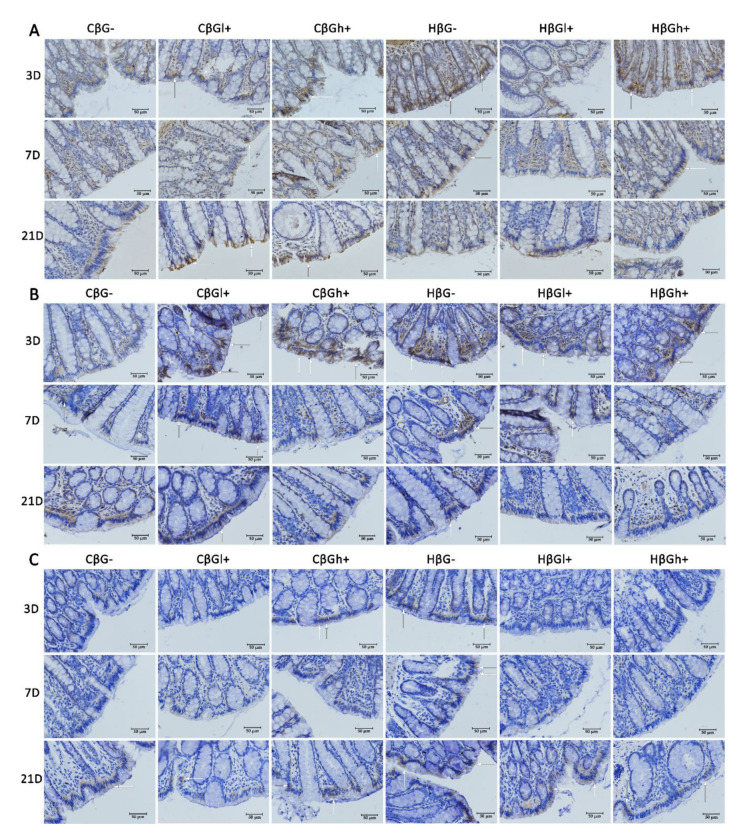

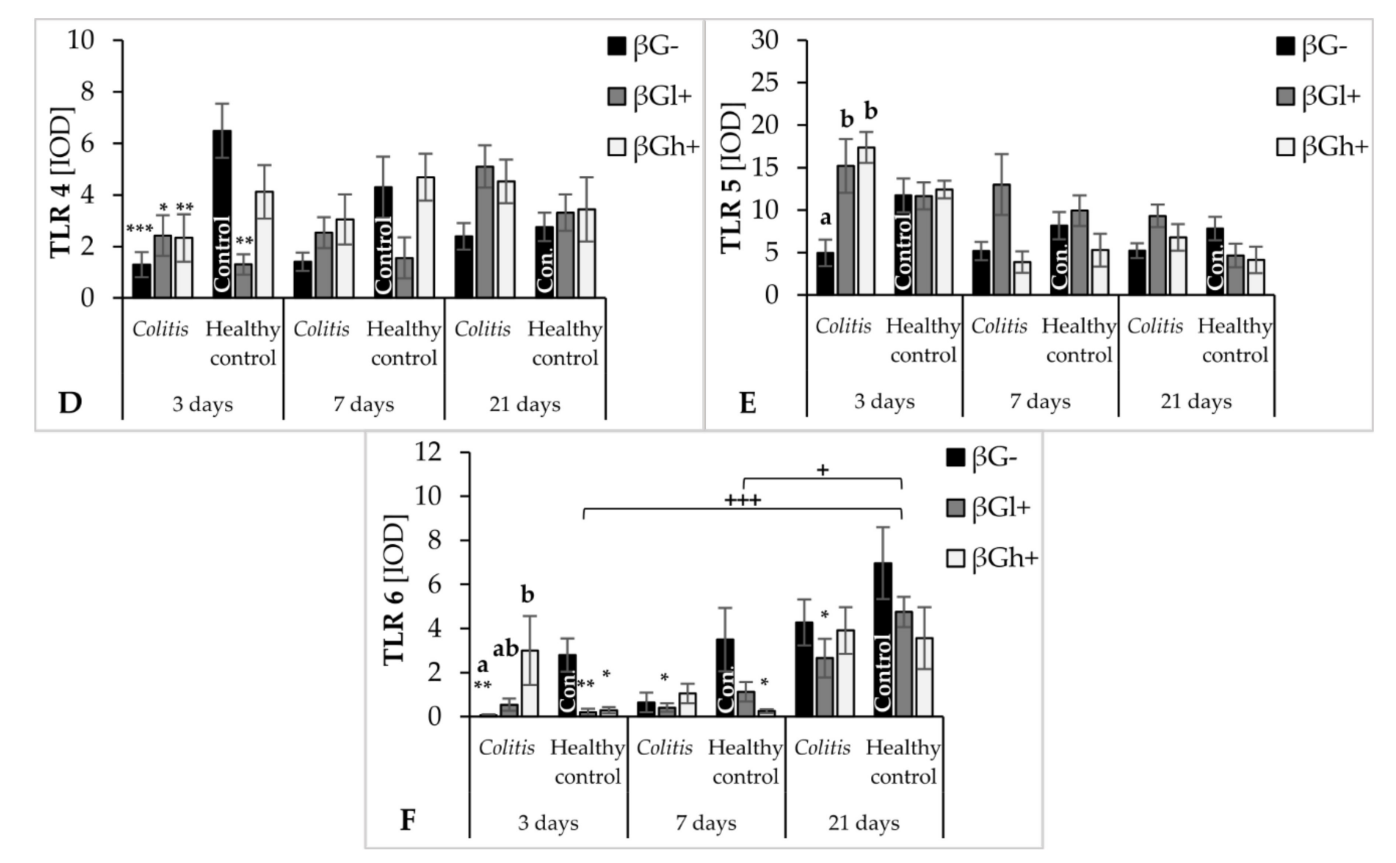
Expression of Toll-like receptors (TLRs) in the colonocytes: results of the immunohistochemical analysis. White arrows indicate colonocytes with high expression of the TLRs (brown precipitate). (**A**–**C**)—Light micrographs imaged under the NIKON Eclipse Ti2 microscope (×40 magnification). (**D**–**F**)—Changes in the expression of TLRs (TLR 4, TLR 5, and TLR 6, respectively) presented (mean ± SE) as integrated optical density (IOD). TLR 4 antigen is represented by a brown precipitate in the colonocytes in (**A**). TLR 5 antigen is represented by a brown precipitate in the colonocytes in (**B**). TLR 6 antigen is represented by a brown precipitate in the colonocytes in (**C**). * Significantly different from the control group (healthy control βG−) at the same time point according to the Dunnett post hoc test (* *p* < 0.05, ** *p* < 0.01, *** *p* < 0.001). ^+^ Significantly different from the same subgroups at another time point according to the Tukey post hoc test (^+^ *p* < 0.05, ^+++^ *p*<0.001). ^a,b^ Different letters denote significant differences in the *colitis*/healthy control group at the same time point according to the Tukey post hoc test (*p* < 0.05).

Changes in the expression of TLRs are presented in Figure 4. ANOVA showed a significant interaction between two experimental factors: induced inflammation of the colon and feed supplementation with oat beta-glucans, which influenced the expression level of all the examined TLRs (ANOVA, *p* < 0.001; Figures S2A, S3C and S4C)—TLR 4, TLR 5, and TLR 6. Post hoc analysis showed a significantly lower expression of TLR 4 and TLR 6 in rats from the *colitis* group that consumed feed without beta-glucans (CβG−) compared to the control rats receiving the same feed (HβG−) (Tukey post hoc, *p* < 0.01). In the case of TLR 5, a significantly lower expression was found only in the CβG− group compared to the CβGl+ group (Tukey post hoc, *p* < 0.001).

Statistical analysis also revealed that TLR 4 expression significantly differed between the subgroups only 3 days after *colitis* induction (Figure 4A,D), while a significantly lower expression was found in all *colitis* subgroups compared to the control group (HβG−) (Dunnett post hoc, *p* < 0.001), with the greatest differences observed between the CβG− and HβG− groups. It should be noted that the expression of this protein was also significantly lower in the control group that consumed the feed with low-molar-mass beta-glucans (HβGl+) compared to the rats from the HβG− group. The effect of the dietary intervention length and inflammation on the TLR 4 expression was confirmed by ANOVA (*p* < 0.05; Figure S2B), which showed an increase in the expression of this receptor in the *colitis* group and a decrease in differences observed between the *colitis* groups and the control groups at all time points after the induction of large-intestine inflammation.

The expression of TLR 5 was influenced by the time after TNBS administration and feed supplementation with oat beta-glucans (Figure 4B,E). ANOVA showed a significant decrease in the expression of this receptor parallel to the extension of the post-TNBS period (ANOVA, *p* < 0.0001; Figure S3A). The highest expression of TLR 5 was found in rats fed with feed supplemented with low-molar-mass oat beta-glucans, while the lowest expression was found in the group of animals fed with feed without beta-glucans (ANOVA, *p* < 0.01; Figure S3B). Post hoc analysis showed significant differences between the experimental groups only after 3 days of TNBS administration with a lower expression observed in the *colitis* group fed with feed without beta-glucans (CβG−) compared to the *colitis* groups treated with low- and high-molar-mass beta-glucans (CβGl+ and CβGh+) (Tukey post hoc, *p* < 0.05).

TLR 6 expression differed significantly between the dietary groups (Figure 4C,F). One-way ANOVA showed a significant effect of TNBS administration (ANOVA, *p* < 0.0001; Figure S4A) and consumption of feed with or without oat beta-glucans (ANOVA, *p* < 0.05; Figure S4B). After 21 days of induction of colon inflammation, the expression of TLR 6 was approximately four times higher compared to that after 3 or 7 days. The highest expression of this receptor was found in the group of rats fed with feed without oat beta-glucans. ANOVA revealed a statistically significant interaction between three experimental factors: inflammation, length of period after TNBS administration, and the type of feed (ANOVA, *p* < 0.001). Three days after TNBS administration, a significantly lower expression of TLR 6 was noted in CβG−, HβGl+, and HβGh+ groups compared to the control group (HβG−) (Dunnett post hoc, groups CβG− and HβGl+ vs. group HβG−: *p* < 0.01; group HβGh+ vs. group HβG−: *p* < 0.05). Seven days after the induction of colitis, a lower expression of this receptor was found only in the CβGl+ and HβGh+ groups compared to the HβG− group (Dunnett post hoc, *p* < 0.05). Twenty-one days after the induction of colitis, TLR 6 expression differed significantly only between CβGl+ and HβG− groups (Dunnett post hoc, *p* < 0.05). It should be added that TLR 6 expression in the *colitis* group fed with feed supplemented with high-molar-mass beta-glucans (CβGh+) was at a similar level as in the control group (HβG−) group after 3, 7, and 21 days of TNBS administration.

### 3.3. Autophagy and Apoptosis Markers Expression in the Large Intestine

**Figure 5:**
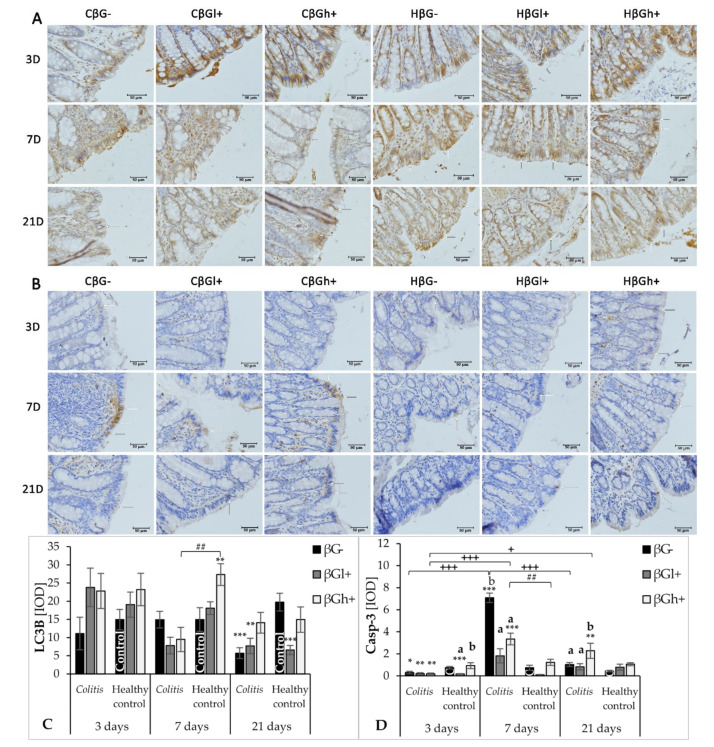
Expression of autophagy and apoptosis markers in the colonocytes: results of the immunohistochemical analysis. White arrows indicate colonocytes with high expression of the LC3B protein (**A**) and Caspase-3 protein (**B**) (brown precipitate). (**A**,**B**)—Light micrographs imaged under the NIKON Eclipse Ti2 microscope (×40 magnification). (**C**,**D**)—Changes in the expression of autophagy and apoptosis markers (LC3B and Caspase-3 (Casp-3), respectively) presented (mean ± SE) as integrated optical density (IOD)**.** Autophagy-related protein LC3B antigen is represented by a brown precipitate in the colonocytes in (**A**). Apoptosis-related protein Casp-3 antigen is represented by a brown precipitate in the colonocytes in (**B**). * Significantly different from the control group (control βG−) at the same time point according to the Dunnett post hoc test (* *p* < 0.05, ** *p* < 0.01, *** *p* < 0.001). ^#^ Significantly different between the *colitis* and control groups at the same time point and the same feed according to the Tukey post hoc test (^##^ *p* < 0.01). ^+^Significantly different from the same subgroups at another time point according to the Tukey post hoc test (^+^ *p* < 0.05, ^+++^ *p*<0.001). ^a,b^ Different letters denote significant differences in the *colitis*/control group at the same time point according to the Tukey post hoc test (*p* < 0.05).

Immunohistochemical results showing the expression of selected markers of autophagy (LC3B) and apoptosis (Caspase-3) in IECs are presented in Figure 5. ANOVA showed a significant influence on LC3B expression by all three experimental factors: time since TNBS administration, consumption of oat beta-glucans with feed, and the occurrence of inflammation. Analyzing each factor separately, it was found that LC3B expression decreased with time since TNBS administration (ANOVA, *p* < 0.001; Figure S5A), as well as a significant reduction in its expression, was caused by the induction of colitis (ANOVA, *p* < 0.01; Figure S5C). The consumption of feed with high-molar-mass oat beta-glucans resulted in a significantly higher expression of LC3B compared to other dietary groups (ANOVA, *p* < 0.05; Figure S5B). ANOVA also showed a significant interaction between the three experimental factors (*p* < 0.01), which was reflected by a significantly higher expression of LC3B after 7 days in the HβGh+ group compared with the CβGh+ group (Tukey post hoc, *p* < 0.01), while after 21 days, the expression of this protein was significantly lower in the CβG−, CβGl+, and HβGl+ groups compared to the control group (HβG−) (Dunnett post hoc, *p* < 0.001, *p* < 0.01, and *p* < 0.01, respectively).

All three investigated factors significantly influenced the expression of Caspase-3 (ANOVA, *p* < 0.0001; Figure S6A–C). The expression of this apoptotic protein 3 days after TNBS administration was at a very low level, but it was significantly lower in the *colitis* groups and HβGl+ group compared to the control group (HβG−) (Dunnett post hoc, CβGl+ vs. HβG−: *p* < 0.05; CβGh+ and CβG− vs. HβG−: *p* < 0.01; HβGl+ vs. HβG−: *p* < 0.001), while in the HβGh+ group it was at the same level as in the HβG− group. After 7 days of the experiment, the expression of Caspase-3 in the *colitis* group fed with feed without beta-glucans (CβG−) in relation to other experimental groups was very high, while a significantly lower expression was found in the *colitis* subgroups fed with feed supplemented with both forms of beta-glucans (CβGl+ and CβGh+) compared to the CβG− group (Tukey post hoc, *p* < 0.05). However, a very low expression of this enzyme was observed in all control groups. Caspase-3 expression in the CβGh+ group after 21 days was at a similar level as the expression found after 7 days, with a significantly higher level compared to other *colitis* groups (Tukey post hoc, *p* < 0.05) and control group (HβG−) (Dunnett post hoc, *p* < 0.01).

**Figure 6:**
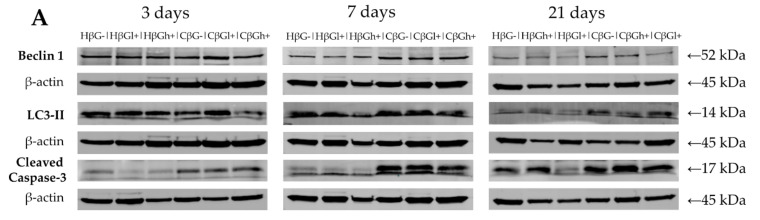

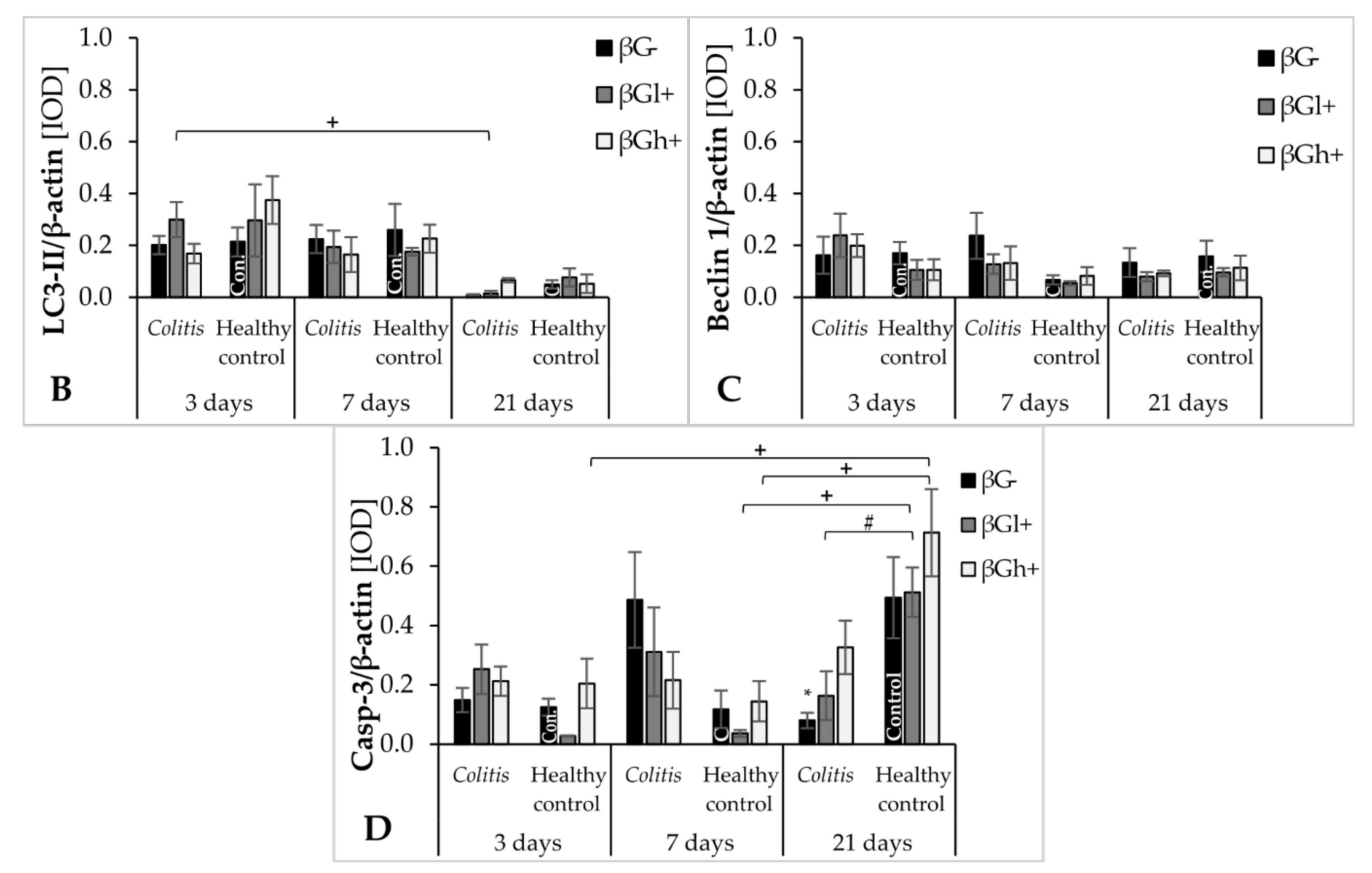
Results of the densitometric analysis for the expression of autophagy and apoptosis markers in the large intestinal wall. (**A**)—Representative immunoblot images. (**B**)—Autophagy-related protein LC3-II. (**C**)—Autophagy-related protein Beclin 1. (**D**)—Apoptosis-related protein cleaved Caspase-3 (Casp-3). * Significantly different from the control group (healthy control βG−) at the same time point according to the Dunnett post hoc test (* *p* < 0.05). ^#^ Significantly different between the *colitis* and control groups at the same time point and the same feed according to the Tukey post hoc test (^#^ *p* < 0.05). ^+^ Significantly different from the same subgroups at another time point according to the Tukey post hoc test (^+^ *p* < 0.05).

Figure 6 shows the changes in the expression of autophagy- (LC3-II and Beclin-1) and apoptosis-related proteins (Caspase-3) in the colon wall determined by Western blot. As the results of ANOVA indicate, only time elapsed after TNBS administration had a significant effect on the reduction of LC3-II expression, while the induction of inflammation had a significant effect on Beclin-1 expression with a higher expression observed in the *colitis* group (ANOVA, *p* < 0.05; Figure S7). However, post-hoc analysis showed no significant differences between the experimental subgroups.

After 21 days of TNBS administration, Caspase-3 expression was found to be significantly higher (ANOVA, *p* < 0.001; Figure S8A). Two-way ANOVA showed significant interactions between the time since TNBS administration and induced inflammation (ANOVA, *p* < 0.001; Figure S8B). After 7 days of colitis induction, a significantly higher expression of Caspase-3 in the colon wall was found in the *colitis* group (Tukey post hoc, *p* < 0.05), and after 21 days after colitis induction, a higher expression was observed in the noninflammatory group (Tukey post hoc, *p* < 0.001). Post hoc analysis confirmed a significantly higher expression of this enzyme 21 days after TNBS administration in HβGl+ and HβGh+ groups compared to the expression observed in the corresponding *colitis* subgroups after 7 days of TNBS administration (Tukey post hoc, *p* < 0.05), while it confirmed a lower expression in the *colitis* group that consumed feed without beta-glucans (CβG−) compared to the control group that consumed the same feed (HβG−) (Dunnett post hoc, *p* < 0.05).

### 3.4. Fisher’s Linear Discriminant Analysis (FLD)

**Figure 7:**
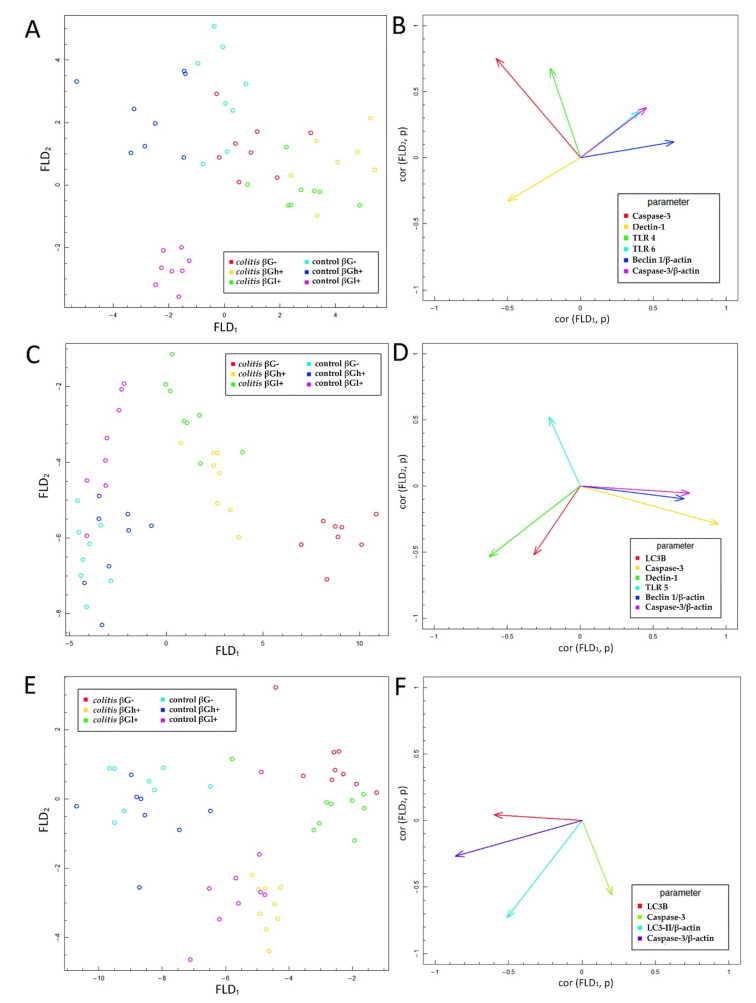
Fisher’s linear discriminant (FLD) analysis. (**A**,**C**,**E**)—Experimental data on the plane spanned by two of the most data-separating FLDs. (**B**,**D**,**F**)—Parameters contributing the most to the FLDs. Scheme (**A**,**B**): 3. days; (**C**,**D**): 7 days; (**E**,**F**): 21 days.

The results of the FLD analysis obtained for the expression of experimental factors are presented in Figure 7. This analysis was used to find the linear combinations of expression of the analyzed receptors and autophagy and apoptosis markers which allow for the best separation of the groups of animals at three time points selected in the experimental model. The experimental data were divided into three groups depending on the experimental time point, as well as the stage of inflammation corresponding to different periods of the disease (exacerbation and remission period). The FLD method provides the most optimal linear combination of parameters used in the analysis such that the highest possible separation between the data groups is achieved. The results of the linear discriminant analysis at three time points are shown in Figure 7A,C,E. In each figure, six experimental groups are isolated. The data are presented in the space between the linear combinations of parameters (FLDs), marked as FLD_1_ and FLD_2_, which separate the best-predefined groups.

Figure 7B,D,F shows the correlation vectors of the analyzed parameters in the experiment with FLD_1_ and FLD_2_ predictors, which determine the direction and strength of the separation of the experimental groups at three time points—3 days (A and B), 7 days (C and D), and 21 days (E and F). The graphs show the parameters that had the greatest impact on the separation of data at particular time points after TNBS administration. The parameter corresponding to a particular vector caused the data to shift in the direction determined by the vector. The performed FLD analysis complemented the ANOVA models by indicating the common set of features that separates the best experimental data (as opposed to the analysis of individual features in the ANOVA model) and allows for an augmented analysis of the obtained results.

FLD analysis performed for the data 3 days after TNBS/saline administration (Figure 7A,B) showed that the factors that most differentiated these experimental groups were Caspase-3 (determined in colonocytes as well as in the whole colon wall), immunohistochemical expression of Dectin-1, TLR 4, and TLR 6 in colonocytes, and expression of Beclin-1 autophagy protein in the colon wall. The FLD analysis allowed determining the combination of the above parameters, which in turn helped in distinguishing the control feed groups supplemented with beta-glucans (HβGl+ and HβGh+) from the other groups in the horizontal plane (FLD_1_). The vertical plane (FLD_2_) enabled the separation of the control group fed with feed supplemented with low-molar-mass oat beta-glucans (HβGl+) from the other groups (Figure 7A). The expression of Beclin-1 protein was the most correlated with FLD_1_, while the expression of TLR 4 and Caspase-3 in colonocytes had the most influence on FLD_2_, and that of Dectin-1, TLR 6, and Caspase-3 in the colon wall was important for both FLDs (Figure 7B). Based on the linear discriminant analysis, it can be concluded that the control group supplemented with βGl+ was characterized by a significantly higher expression of Dectin-1 receptor and a lower expression of TLR 6 receptor and Caspase-3 protein in the colon wall compared to the *colitis* groups. The highest Caspase-3 expression was observed in the control group fed with feed supplemented with high-molar-mass oat beta-glucans (HβGh+).

After 7 days of *colitis* induction (Figure 7C,D), the separation of the *colitis* group from control groups was much more visible than after 3 days. The horizontal plane (FLD_1_) allowed clear separation of control groups from the *colitis* groups, as well as the separation of *colitis* group (CβG−) and *colitis* groups that consumed the feed supplemented with beta-glucans (CβGl+ and CβGh+). Animals from the groups fed feed with a low-molar-mass oat beta-glucans (HβGl+ and CβGl+) were separated from the other groups, in particular from those receiving feed without beta-glucans supplementation (HβG− and CβG−), also in the vertical FLD direction (FLD_2_), however to a lesser extent. Expression of Caspase-3 (in colonocytes and colon wall) and Beclin-1 in the colon wall had the greatest influence on FLD_1_, while TLR 5 expression had the most impact on FLD_2_. Both FLDs were influenced by the expression of Dectin-1 receptor and LC3B protein in colonocytes. Therefore, it can be concluded that the *colitis* group fed with feed without beta-glucans had the highest expression of Caspase-3 and Beclin-1, while the lowest expression of both these proteins was observed in the control groups. In addition, HβG− and HβGh+ groups showed the highest expression of Dectin-1 receptor and LC3B protein. The highest TLR 5 expression was found in the βGl+-fed groups, both with and without *colitis*.

After 21 days of TNBS administration (Figure 7E,F), the analyzed parameters differentiated the experimental groups to a much lesser extent than at earlier time points (3 and 7 days). After 21 days of *colitis* induction, only the expression of LC3B/LC3-II and Caspase-3 in the colonocytes and colon wall allowed separating the experimental groups. CβG− group together with CβGl+ group was separated by FLD_1_ from other subgroups, while HβGl+ and CβGh+ groups were separated from other subgroups along the vertical axis (FLD_2_). LC3B expression in colonocytes and Caspase-3 expression in the colon wall had the greatest impact on FLD_1_, whereas Caspase-3 expression in colonocytes influenced FLD_2_. Expression of LC3-II in the colon wall had an effect on both FLDs (Figure 7F). FLD analysis showed that LC3B expression in the colonocytes and that of Caspase-3 in the colon wall were slightly higher in the control groups fed with βGh+ and βG− feed compared to the CβGl+ and CβG− groups. Expression of Caspase-3 in colonocytes and that of LC3-II in the colon wall were slightly higher in the HβGl+ and CβGh+ groups compared to the CβG− and CβGl+ groups.

## 4. Discussion

Crohn’s disease belongs to the group of inflammatory bowel diseases and is characterized by the presence of chronic inflammation within the gut. The course of this disease is associated with alternating periods of exacerbation and remission. Food is one of the significant factors influencing the course of CD through many mechanisms. Another influencing factor is the modulation of cellular processes, including cell death, which maintains homeostasis and eliminates abnormal and damaged cells [2]. In this study, we determined the effects of the consumption of low- and high-molar-mass oat beta-glucans on the expression of selected markers of apoptosis and autophagy in colonocytes in TNBS colitis-induced rats. In addition, we analyzed the expression of colon wall receptors, including TLRs and Dectin-1, which are involved in the recognition of molecular patterns of pathogens in colon epithelial cells. Rectal administration of a TNBS ethanol solution in animals caused transmural colitis, which is a well-described intestinal inflammation animal model with predominantly Crohn’s disease symptoms [31,33]. According to Antoniou and coworkers, TNBS-induced colitis is a good model for studying immunopathogenesis and potential treatments for Crohn’s disease [30]. In this model intestinal inflammation is achieved by a local administration of TNBS in 50% ethanol which involves both chemical damage and T cell immune reactivity. Additionally, TNBS administration results in acute necrosis of the colon wall due to oxidative damage, along with transmural inflammation that closely resembles the histopathological lesions developed in human CD [30,34,35]. The ethanol breaks the mucosal barrier allowing the penetration of the reagent. The main symptoms in animals with TNBS-induced colitis are bloody diarrhoea, weight loss and intestinal wall thickening. The presence of the features typical of CD was confirmed by histological evaluation, which revealed widespread inflammation of the colon, including not only the mucosa but also the deeper layers of the intestinal wall. The induction of acute inflammation in the colon wall was also confirmed by the results obtained from the analysis of the concentration of proinflammatory cytokines and the lymphocyte profile performed on the same biological material [28] as well as by the results of pro-inflammatory cytokines gene expression. Here, the effects of beta-glucans were assessed at three time points: after 3, 7, and 21 days of TNBS administration. This allowed evaluating the analyzed parameters in animals with different intensities of inflammatory changes in the intestine. It also reflects the periods of exacerbation and remission of colitis in people with CD and enabled determining the effectiveness of beta-glucan supplementation in these periods of the disease. The examined rats developed local acute inflammation immediately after TNBS administration. The inflammation was significantly less severe 1 and 3 weeks after TNBS administration, which indicated remission of the induced inflammation. This was confirmed by macro- and microscopic examinations of changes in the colon, including swelling of the mucosa, microbleeding, and necrosis [28]. It should be noted that the changes were transient and minimally invasive, indicating an early stage of CD development. This was evidenced by the good health of rats, decreased feed consumption only five days after the administration of TNBS, lower weight gain in rats with induced colitis, and no increase in the activity of plasma liver transaminases [29].

In this study, we also examined changes in the intensity of the autophagy process. It contributes to the adaptation of cells and the maintenance of intracellular homeostasis enabling cells to survive under stressful conditions. The autophagy marker in the colon wall and IECs investigated in the study was the expression of the LC3B protein, which participates in the formation and maturation of autophagosomes [8]. A decrease in this protein was found in the colon wall after TNBS administration, which indicates intense repair processes of the intestinal epithelium accompanying/preceding the period of CD remission. In the initial period of the experiment, autophagy was slightly increased in the colon wall in all experimental groups, which could be due to the mechanical irritation caused by rectal administration of TNBS or saline solution. A significant effect of colitis on the reduction of LC3B expression in IECs was found after 21 days. At this time point, the expression of LC3B in colonocytes was approximately four times lower in the colitis rats fed with feed without beta-glucans (CβG−) compared to the βG− control group. A similar effect of colitis was observed in induced rats by Xiong et al. (2019), who showed a decrease in the expression of LC3-II in the epithelium of the large intestine in colitis-induced animals compared to the control group [36]. In humans, CD is also characterized by impaired autophagy [37]. A study recognized polymorphism in the ULK1 kinase gene involved in the autophagy process and mutation of the protein containing the nucleotide-binding oligomerization domain 2 (NOD2) as a direct mechanism responsible for the autophagy impairment in CD [8].

Our results showed that supplementation with high-molar-mass oat beta-glucans reduced the negative effects of inflammation on the expression of LC3B protein. After 21 days of the experiment, it was observed that the expression of LC3B in IECs in the colitis group fed with feed supplemented with high-molar-mass oat beta-glucans was similar to that in the βG− control group. The increase in the expression of this protein under the influence of high-molar-mass beta-glucans in animals without colitis after 7 days of the experiment was also significant compared to other experimental groups. This may indicate the autophagy-enhancing effect of these polysaccharides in IECs in animals with and without colitis. The results of a previous study conducted by our team also indicated the effect of supplementing animal feed with oat beta-glucans on the expression of autophagy-related proteins. In an experiment carried out on rats in which enteritis was induced by intravenous administration of LPS, the transcriptomic analysis showed that the consumption of oat beta-glucans with feed increased the expression of the *Atg10* gene belonging to the *ATG* (autophagy-related genes) family. In addition, high-molar-mass oat beta-glucans caused a significant upregulation of the expression of the *Atg13* gene, another gene of the *ATG* family [38]. Therefore, the above data indicate a significant effect of oat beta-glucans in restoring the activity of the autophagy process in inflamed IECs, and a stronger effect of oat beta-glucans with a high molar mass, which also increased the activity of autophagy in colon tissue of the control animals without colitis.

Disturbances in the apoptotic process are believed to play an important role in the pathogenesis of CD. On one hand, excessively intensified apoptosis of epithelial cells leads to their increased elimination and damage to the intestinal barrier [39]. On the other hand, the low intensity of apoptosis of inflammatory cells promotes their accumulation in the gastrointestinal wall and maintenance of inflammation and promote tumor progression [24,40]. The present study showed that in the early stages of the development of acute colitis in animals with colitis, the expression of Caspase-3, the executive enzyme of apoptosis, was very low. It indicates a strong inflammation caused by the rectal administration of the TNBS ethanol solution. The highest expression of Caspase-3 protein was observed in the control group fed with feed supplemented with high-molar-mass oat beta-glucans (HβGh+), which indicates the beneficial effect of this beta-glucan fraction in this respect. The physical properties of high-molar-mass beta-glucans favor the formation of a protective layer on the inner wall of the intestine, and hence this beta-glucan fraction effectively supports the development of beneficial microbiota producing short-chain fatty acids which favor the faster regeneration of epithelium damaged by saline administration [23,41,42].

After 7 days of TNBS administration, Caspase-3 expression in colitis-induced animals was approximately eight times higher than in the control group, which indicates an increase in the apoptosis process in response to intestinal inflammation. This was also confirmed by the FLD analysis, in which the control groups were clearly separated from the colitis groups. The clear separation of the control groups was mainly due to the greater Caspase-3 expression in the colon epithelium and colon wall in inflammatory animals. An intensified process of apoptosis is also observed in people with CD and UD. This is associated with the disturbance of the intestinal barrier integrity, which causes the transfer of the commensurate microbiota to the intestinal lamina propria. This in turn leads to an increase in inflammation and the level of proinflammatory cytokines such as TNF-α [9,43]. Oat beta-glucans may reduce the extent of apoptosis in the colon tissue and, consequently, improve the integrity of the intestinal barrier. In our study, consumption of feed supplemented with high-molar-mass oat beta-glucans by colitis animals resulted in approximately two times lower Caspase-3 expression after 7 days of inflammation induction. Consumption of feed with low-molar-mass oat beta-glucans resulted in the expression of this enzyme in colitis animals at a similar level as in the control group, which was also confirmed by the results of the FLD analysis. This proves that both forms of beta-glucans are effective in inhibiting apoptotic cell death, reducing inflammation markers, and inducing the remission period. In colitis rats fed with feed supplemented with oat beta-glucans of high molar mass after 7 and 21 days, the expression level of Caspase-3 was similar, but after 21 days it was higher compared to the other feeding groups. This confirms the significant effect of oat beta-glucans with a low and high molar mass in reducing the expression of this apoptotic enzyme during ongoing inflammation, with low-molar-mass beta-glucans having a stronger effect.

Oat beta-glucans influence the activity of the autophagy process and inhibition of apoptosis in inflamed IECs. It is probably related to the interaction of oat beta-glucans on the Dectin-1 receptor and the pattern recognition TLRs: TLR 4, TLR5, and TLR6. These receptors are responsible, among others, for the stimulation of the immune response to pathogenic factors, as well as the regulation of proliferation, autophagy, and apoptosis of intestinal cells [15,17]. In our experiment, the expression of Dectin-1 across all experimental time points was found to be reduced compared to the control group, due to the induced inflammation. It should be noted, that de Vries et al. showed increased Dectin-1 expression in an animal model of DSS-induced colon and mesenteric lymph nodes inflammation [44], while Van Hung and Suzuki showed no significant changes in the expression of this receptor in the DSS-induced small intestine inflammation [45]. Similarly, in patients with Crohn’s disease, increased expression of the Dectin-1 receptor in macrophages, neutrophils, and other immune cells has been reported [46]. The observed inconsistency is likely caused by the differences in colitis induction and the type of cells examined for Dectin-1 expression assessment. According to our knowledge, there are no reports describing the effect of TNBS-induced colitis on Dectin-1 expression in intestinal epithelial cells. The decrease in Dectin-1 expression observed in our study may result from a different method of inducing colitis. In addition, we analyzed the Dectin-1 expression in the intestinal epithelial cells, not in the colon wall or the immune cells. In this case decrease in Dectin 1 expression in colonocytes noticed in our study may be related to disrupted intestinal barrier integrity by the ethanolic TNBS solution, that as a consequence, causes infiltration of pathogens/antigens into the deeper layers of the colon wall and allows their direct contact with cells of the immune system. The subsequent activation of immune cells results in an increased expression of Dectin-1 in these cells [30,47]. Moreover, in our study, it is important to point that the increase in the expression of this receptor in IECs was observed in the control groups after 3 and 7 days of saline solution administration, which could have been caused by mechanical damage to these cells evidenced by the lack of similar changes after 21 days. This confirmed that the administration of the saline solution did not cause the injury of the intestinal barrier, that was observed after TNBS administration. We also observed simultaneous reduction of colonocyte Dectin-1 in the colitis group and its increase in control groups caused by the mechanical stimulus that resulted in a significant difference in the expression of this receptor between these groups. Consumption of the feed supplemented with oat beta-glucans reduced the difference between colitis and control groups in the experimental animals on days 3 and 21 after TNBS administration. The results showed an increase in the expression of Dectin-1 in inflamed IECs under the influence of oat beta-glucans. It should be noted that these polysaccharides have a similar effect on the expression of LC3B protein, with oat beta-glucans with a high molar mass having a stronger effect. FLD analysis showed that after 7 days of TNBS administration, the increased expression of Dectin-1 receptor was accompanied by an increased expression of the LC3B protein, the increase being characteristic of the control group fed with feed without beta-glucans and the control group fed with feed with beta-glucans with a high molar mass, which indicates the intensification of autophagy processes stimulating epithelial repair. Sakaguchi et al. (2018) described autophagy activation caused by the attachment of polysaccharide ligands to the Dectin-1 receptor as the mechanism of action of fungal beta-glucans administered to mice with induced colitis. These authors demonstrated that administration of beta-glucans decreased the expression of inflammatory cytokine mRNA in the colon (*Tnf*, *Il1b*, *Il6*) and the expression of TNFR1 receptor in IECs through the interaction of these polysaccharides with the Dectin-1 receptor. In addition, activation of Dectin-1 by beta-glucans reduced the translocation of NF-κB responsible for the induction and maintenance of inflammation [21].

Our other results showed that the concentration of TNF-α and other proinflammatory cytokines in the colon wall of animals with TNBS-induced inflammation was significantly increased at all time points. Consumption of oat beta-glucans reduced the concentration of these inflammatory factors (data not published [28]). TNF-α is one of the inflammatory mediators that directly stimulate the apoptotic process. The extrinsic pathway of apoptosis is induced by the binding of this factor to the TNFR1a receptor [48]. Pott et al. (2018) showed that increased autophagy in the inflamed intestinal epithelium protected cells against TNF-α-induced apoptosis, which in turn helped to maintain the integrity of the intestinal barrier and reduce inflammation. Thus, autophagy disorders cause exacerbation of inflammation and intensification of apoptosis in IECs induced by proinflammatory cytokines, mainly TNF-α [39]. This is in line with the results of another study in which the authors used a model of enteritis induced by murine norovirus infection and administration of DSS (dextran sulfate sodium). Matsuzawa-Ishimoto et al. (2017) showed that autophagy deficiency caused by deletion of the *ATG16L1* gene (encoding a component of the large protein complex essential for autophagy) made IECs in the small intestine more susceptible to TNF-α-induced apoptosis [49]. Currently, the treatment of CD in humans is based on drugs that stimulate autophagy and reduce TNF-α concentration in the inflamed parts of the gastrointestinal tract [50]. In summary, the activation of the inflamed Dectin-1 receptor in IECs by oat beta-glucans reduces the proapoptotic effect of the TNF-α complex with TNFR1 receptor. As a consequence of these changes, the autophagy process is intensified, which entails the protection of cells against excessive apoptosis as well as helps to maintain the integrity of the intestinal barrier and alleviate inflammation.

The antiapoptotic effect of oat beta-glucans in colitis is probably also related to their influence on the TLR expression. The results of this study showed that, after 3 days of TNBS administration, the expression of TLR 4 and TLR 6 receptors in colonocytes was significantly lower in the colitis group receiving feed without beta-glucans as compared to the control group fed with the same feed. In addition, in the same group of rats (CβG−), TLR 5 expression was lower compared to the colitis groups fed with feed supplemented with low- and high-molar-mass oat beta-glucans. This difference disappeared with the passage of time after TNBS administration, which proves that the decrease in the expression of these receptors is mostly influenced by acute intestinal inflammation. Nevertheless, oat beta-glucans caused a significant increase in the expression of these receptors, especially TLR 5 and TLR 6, in inflamed IECs in the initial period of the experiment. This period was characterized by the presence of active inflammation. Oat beta-glucans with a low molar mass had a stronger effect in increasing TLR 5 expression, while the expression of TLR 6 was influenced only by oat beta-glucans with a high molar mass.

Deficiency of TLRs, including TLR 4 and TLR 5, increases the susceptibility to induced inflammation resulting in increased permeability of the intestinal epithelium, inhibition of proliferation, increased apoptosis, and delayed IEC differentiation, which in turn leads to increased damage and ineffective reconstruction of the intestinal epithelium [51,52]. On the other hand, activation of TLRs alleviates the symptoms of colitis by stimulating the synthesis of cytoprotective and function-modulating factors in mesenchymal stem cells and immune cells that migrate to the sites of active inflammation. Furthermore, TLRs take part in the repair of the intestinal epithelium by inducing the synthesis of TFF3 (trefoil factor 3), amphiregulin, and prostaglandin E2, which increase the migration, survival, and proliferation of epithelial cells [22]. TLR 4 overexpression in patients with chronic IBD increases the risk of colorectal cancer due to increased proliferation and decreased apoptosis of IECs that are continuously exposed to proinflammatory cytokines and ROS [24,25].

Discriminant analysis by Fisher showed a positive correlation between the expression of TLR 4 and Caspase-3 in colon epithelial cells after 3 days of the experiment. A higher expression of TLR 4 and Caspase-3 was observed in the control groups fed with feed supplemented with high-molar-mass beta-glucans and beta-glucan-free feed compared to all colitis-induced groups, as well as in the control group consuming feed supplemented with low-molar-mass beta-glucans. This may indicate the activation of the apoptotic pathway with TLR 4 participation, which could have been caused by mechanical damage to the intestinal epithelium after rectal administration of physiological saline. Such damage increases the possibility of pathogen contact with the receptors present on the IEC membranes and consequently induces apoptosis. Some results indicate that bacterial LPS, a component of the cell membrane of some pathogenic bacteria, is one of the factors that reduce the proliferation and increase the apoptosis of cells of the small intestine and colon by activating TLR 4 [53,54]. The significant effect of the consumption of low-molar-mass oat beta-glucans by rats from the control group, which showed lower TLR 4 and Caspase-3 expression compared to other control groups, may indicate the antiapoptotic effect of this polysaccharide. As shown by the results of our research, the mechanism of the antiapoptotic action of beta-glucans is associated with decreased expression of TLR 4. It should be noted, however, that the intensity of the apoptosis process was relatively lower in the control groups, especially compared to the colitis groups after 7 days of TNBS administration.

Flagelin is a TLR5-specific ligand and the major protein of the surface structures of bacterial cells. Expression of TLR 5 in IECs regulates the composition and localization of the intestinal microbiota, preventing the development of intestinal inflammation [55]. The intestinal epithelial layer of TLR 5-deficient mice, as indicated in a review by Burgueño and Abreu (2020), was much more colonized by commensal microorganisms. TLR 5 deficiency, accompanied by an abnormal immune response to commensal antigens, led to the spontaneous development of colitis or exacerbation of the existing inflammation in mice [22]. In our study, we observed a significant effect of the consumption of oat beta-glucans on the increase in TLR 5 expression in acutely inflamed IECs, which consequently resulted in equating the expression of this receptor in colitis animals with the expression found in the βG− control group. There are no reports directly linking the effect of beta-glucans with TLR 5 expression. These polysaccharides are not typical ligands for TLRs, so it should be assumed that their likely mechanism of interaction with these receptors is indirect, possibly resulting from the influence on the expression of MyD88 (myeloid differentiation primary response 88) and TRIF (TIR-domain-containing adapter-inducing interferon-β). Studies by other authors have indicated that TLR 5 is an important factor regulating the composition of the intestinal microbiota. However, microbiota does not have a direct impact on the TLR 5 transcript, but on the MyD88 and TRIF adapters, which are among the main signaling molecules modulating the activity of TLRs by promoting epithelial reconstruction and repair as well as the production of cytoprotective factors [51,56].

Interactions between activated Dectin-1 and TLRs also play an important role. As indicated by the results from other authors, the receptors activated by soluble and insoluble beta-glucans, such as Dectin-1, TLR 2, TLR 4, and TLR 6, in macrophage membranes are important modulators of cytokine synthesis and participate in the activation of intracellular metabolic pathways. The main mediator of the activation of Dectin-1-dependent pathways in immune cells is the Syk tyrosine kinase, which, along with the Dectin-1 receptor, activates NF-κB through the CARD9-Bcl10-MALT1 complex [13]. Williams et al. demonstrated that NF-κB1-deficient mice exhibited increased IEC apoptosis in response to TNF-α. This is due to the function of the TNFR1 receptor. In addition to initiating apoptosis, this receptor also induces the expression of antiapoptotic genes by NF-κB [57]. Through their ability to simultaneously interact with the Dectin-1 receptor, TLR 2, and TLR 4, beta-glucans can induce NF-κB in the MyD88 protein-dependent signaling cascade. However, the binding of Dectin-1 by these polysaccharides with a parallel blocked TLR 5 did not have such an effect [13]. These data suggest that TLR 5 binding by beta-glucans does not further modulate the cytokines produced. It should be noted, however, that water-insoluble beta-glucans have a stronger Dectin-1 receptor-activating effect [13]. Such signaling synergy was also demonstrated by Patidar et al., who described the co-localization and clustering of Dectin-1 and TLR 2 in peritoneal macrophages stimulated by barley or yeast beta-glucans (zymosan), with zymosan showing a stronger effect. The phosphorylation pattern in the present study indicates that Dectin-1 and TLR 2 stimulation can activate downstream signaling pathways in a variety of ways. Dectin-1 follows the Syk kinase signaling pathway, while TLR 2 follows the signaling pathway of IκB kinase (IKK-IκB) [58]. Another in vitro study, in which the authors showed increased activation of NF-κB in human dendritic cells by oat beta-glucans via the Dectin-1 receptor, confirms the activation of the Dectin-1/NF-κB signaling pathway by water-soluble beta-glucans. A significant finding in their study was that they noted a stronger stimulatory effect of beta-glucans subjected to enzymatic digestion, which was probably associated with a greater number of β-(1,3) bonds available for Dectin-1 receptors [17].

The mechanism by which beta-glucans influence apoptosis and autophagy in inflamed IECs is related to their effect on the expression of receptors not only in these but also in the cells of the immune system, such as macrophages or dendritic cells. The research results cited above indicate that beta-glucans modulate the synthesis of inflammatory cytokines by the immune cells, which translates into the modification of autophagy and apoptosis signaling pathways in IECs.

In our study, we described for the first time the effects of beta-glucans at three time points—3, 7, and 21 days after TNBS administration, reflecting the periods of exacerbation and remission that occur in people with CD. The results showed significant correlations between the expression of TLRs and Dectin-1 receptors and that of LC3B, Caspase-3, and Beclin-1 protein. Fisher’s discriminant analysis showed such correlations only on days 3 and 7 after induction of colitis, while after 21 days, the analysis showed only the effect of expression of autophagy and apoptosis proteins in the colon wall and epithelium on the differentiation of the experimental groups. The ANOVA and FLD results as well as the results of our previous study indicated a significant effect of time on the effectiveness of beta-glucans in the model of colitis characteristic of CD. In the initial period, the direction of changes in the studied parameters indicates acute local inflammation, while over time, remission occurs [29]. It should be noted that the effect of oat beta-glucans on apoptosis and autophagy through TLRs and Dectin-1 receptors was dependent on the severity of inflammation, which was mainly confirmed by the FLD analysis. With the passage of time after TNBS administration and the progression of inflammatory remission, the immune and metabolic responses to these polysaccharides decreased at the cellular level.

## 5. Conclusions

In summary, oat beta-glucans were found to have the ability to alleviate the course of induced inflammation. Their influence on the course of apoptosis and autophagy seems to be particularly significant. The observed reduction in the activity of apoptosis and increased activity of autophagy, in combination with the immunomodulatory activity of beta-glucans, suggest their beneficial therapeutic effect. Depending on the molar mass, these polysaccharides may act via different signaling pathways, but both consequently reduce inflammation and accelerate remission by, among others, protecting the integrity of the intestinal barrier. The presented results do not clearly indicate which beta-glucan fraction has a more effective protective effect on IECs; however, it seems that oat beta-glucans with a low molar mass have a slightly stronger effect in alleviating the induced colitis by greatly influencing the apoptosis process. In addition, the results published earlier by our team indicated that oat beta-glucans with low molar mass are more effective in removing the systemic effects of colitis, including anti-inflammatory and indirect antioxidant effects [28,29]. These properties of oat beta-glucans, in particular those of low molar mass, indicate their utility as preparations added during the production of food for special medical purposes for people suffering from inflammatory bowel disease, especially Crohn’s disease.

## Data Availability

The data that support the findings of this study are available on request from the corresponding author [K.D.].

## Supplementary Materials

The following are available online at https://www.mdpi.com/2072-6643/13/2/321/s1, Figure S1: DEC-1 Changes expression in colonocytes expressed (mean ± SE) as integrated optical density.; Figure S2: Changes expression TLR 4 in colonocytes expressed (mean ± SE) as integrated optical density.; Figure S3: Changes expression TLR 5 in colonocytes expressed (mean ± SE) as integrated optical density.; Figure S4: Changes expression TLR 6 in colonocytes expressed (mean ± SE) as integrated optical density.; Figure S5: Changes expression LC3B in colonocytes expressed (mean ± SE) as integrated optical density.; Figure S6: Changes expression Caspase-3 in colonocytes expressed (mean ± SE) as integrated optical density.; Figure S7: Results of the densitometric analysis for Beclin 1 expression in the large intestinal wall expressed (mean ± SE) as integrated optical density.; Figure S8: Results of the densitometric analysis for Caspase-3 expression in the large intestinal wall expressed (mean ± SE) as integrated optical density.; Figure S9: Relative expression of cytokine genes vs. Ldha mRNA.
