# Supplementary material for: Effects of Dietary Oat Beta-Glucans on Colon Apoptosis and Autophagy through TLRs and Dectin-1 Signaling Pathways—Crohn’s Disease Model Study
Source: Nutrients. 2021 Jan 22;13(2):321. doi: 10.3390/nu13020321 (PMC7911679; doi:10.3390/nu13020321)
Supplement: Supplementary file 1 [file nutrients-13-00321-s001.pdf]

Supplementary figures:

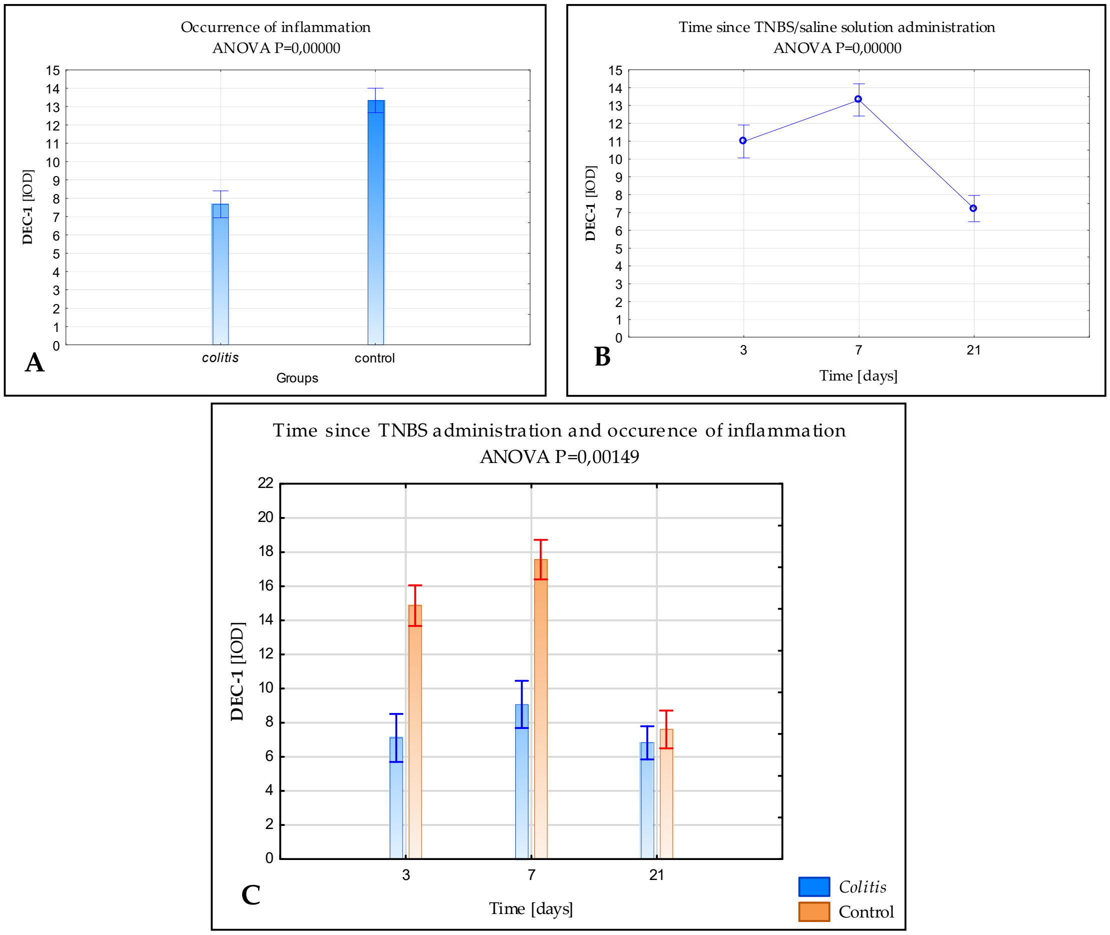

Figure S1. Changes expression **DEC-1** in colonocytes expressed (mean ± SE) as integrated optical density (IOD). ANOVA analysis. **A** – Influence of inflammation; **B** – Influence of time since TNBS/saline solution administration; **C** – Influence of interaction between the time since TNBS/saline solution administration and the occurrence of inflammation.

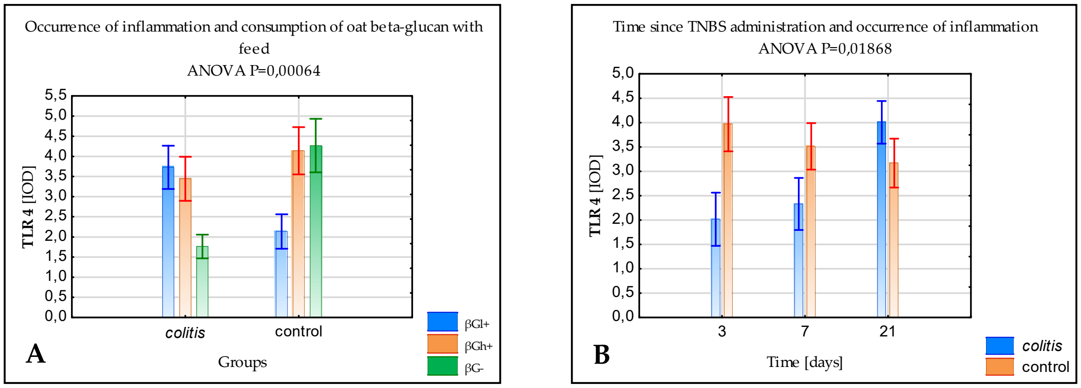

Figure S2. Changes expression **TLR 4** in colonocytes expressed (mean ± SE) as integrated optical density (IOD). ANOVA analysis. **A** – Influence of interaction between the occurrence of inflammation and consumption of oat beta-glucan with feed. **B** – Influence of interaction between the time since TNBS/saline solution administration and the occurrence of inflammation.

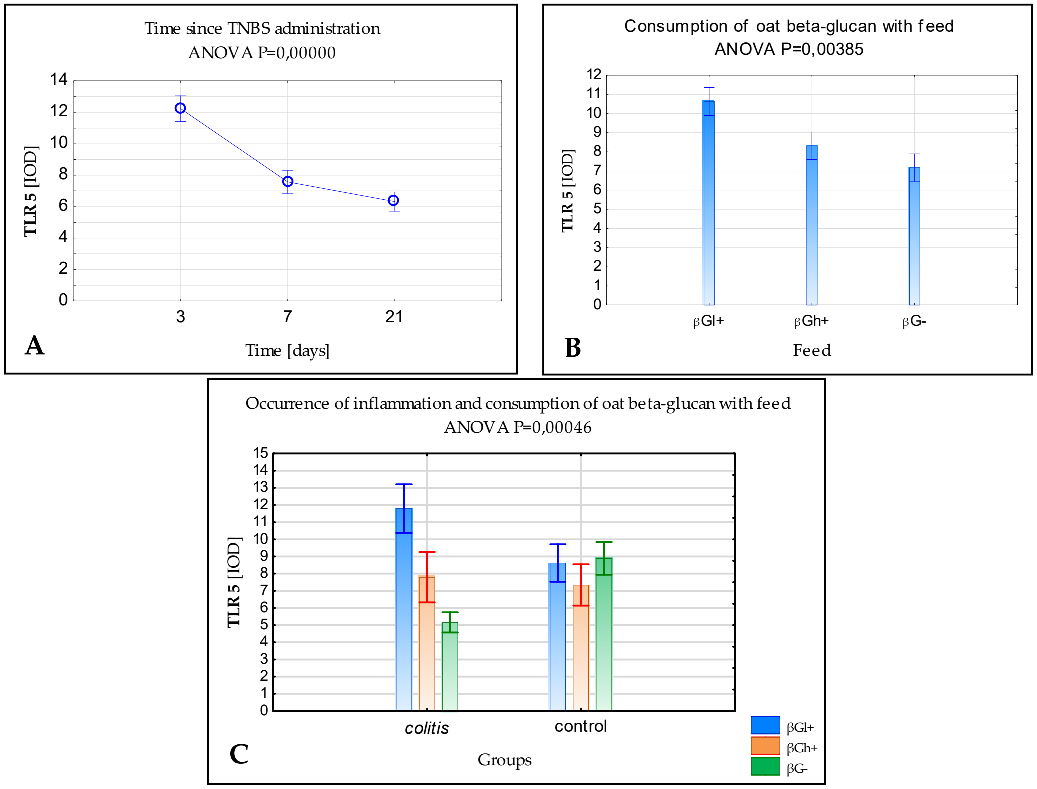

Figure S3. Changes expression **TLR 5** in colonocytes expressed (mean ± SE) as integrated optical density (IOD). ANOVA analysis. **A** – Influence of time since TNBS/saline solution administration. **B** – Influence of consumption of oat beta-glucan with feed. **C** – Influence of interaction between the occurrence of inflammation and consumption of oat beta-glucan with feed.

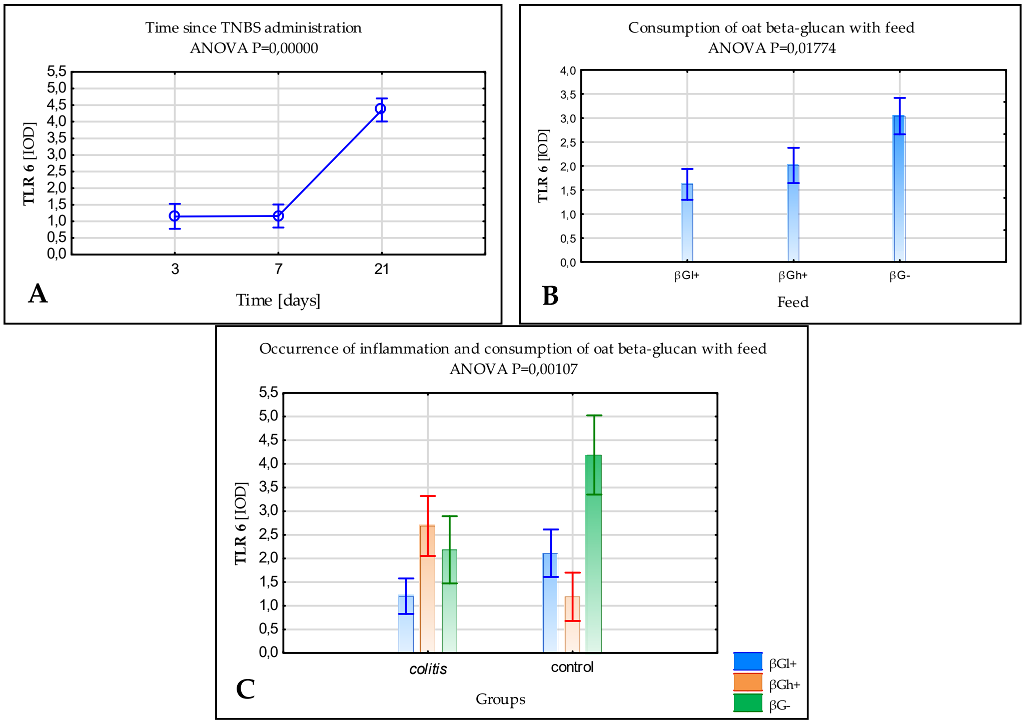

Figure S4. Changes expression **TLR 6** in colonocytes expressed (mean ± SE) as integrated optical density (IOD). ANOVA analysis. **A** – Influence of time since TNBS/saline solution administration. **B** – Influence of consumption of oat beta-glucan with feed. **C** – Influence of interaction between the occurrence of inflammation and consumption of oat beta-glucan with feed.

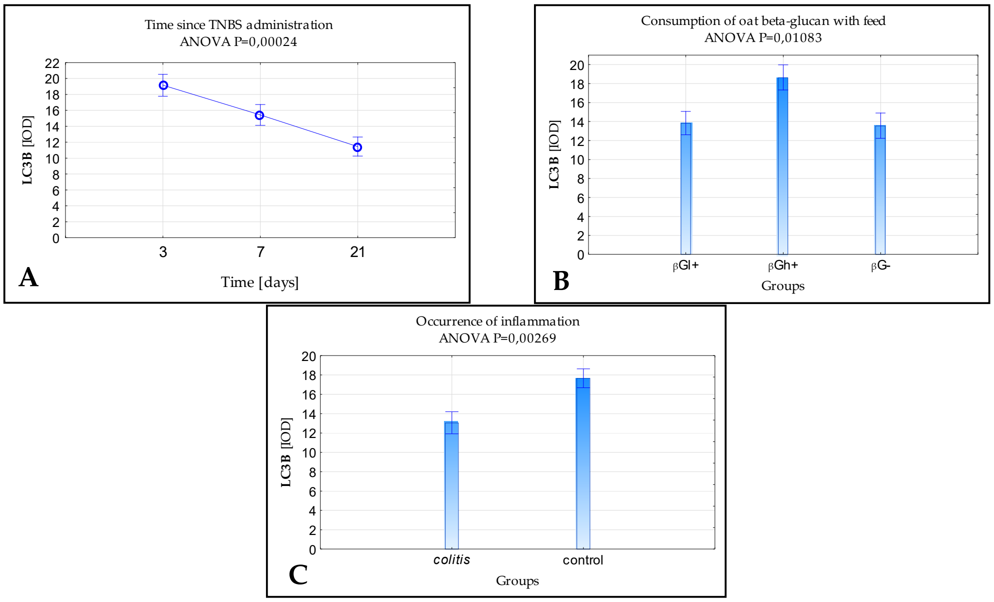

Figure S5. Changes expression **LC3B** in colonocytes expressed (mean ± SE) as integrated optical density (IOD). ANOVA analysis. **A** – Influence of time since TNBS/saline solution administration. **B** – Influence of consumption of oat beta-glucan with feed. **C** – Influence of the occurrence of inflammation.

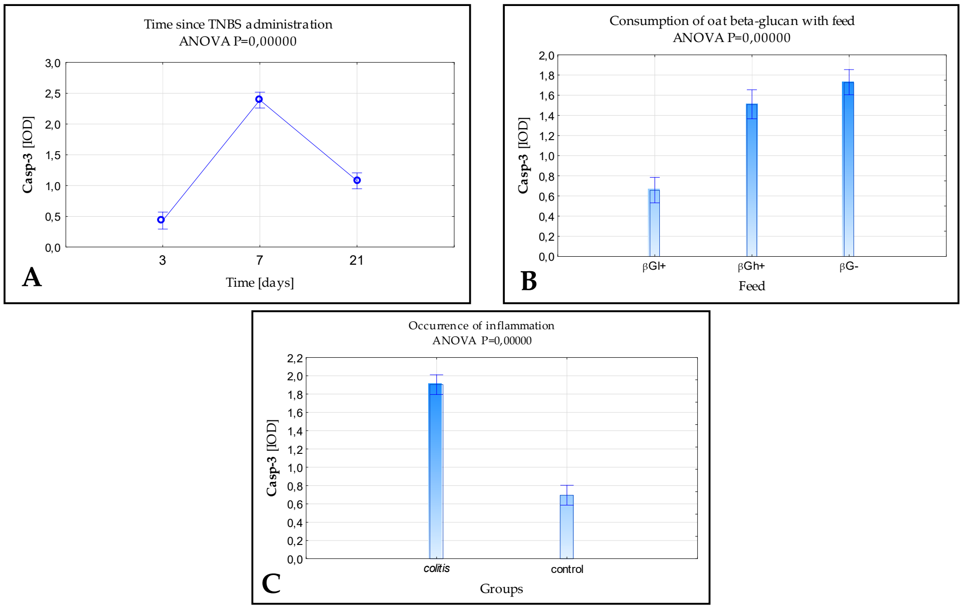

Figure S6. Changes expression **Caspase-3** in colonocytes expressed (mean ± SE) as integrated optical density (IOD). ANOVA analysis. **A** – Influence of time since TNBS/saline solution administration. **B** – Influence of consumption of oat beta-glucan with feed. **C** – Influence of the occurrence of inflammation.

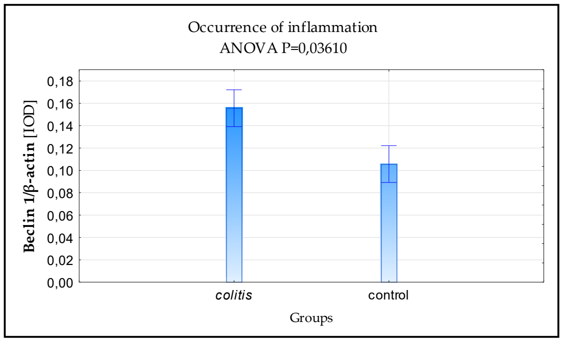

Figure S7. Results of the densitometric analysis for **Beclin 1** expression in the large intestinal wall expressed (mean ± SE) as integrated optical density (IOD). ANOVA analysis. Influence of the occurrence of inflammation.

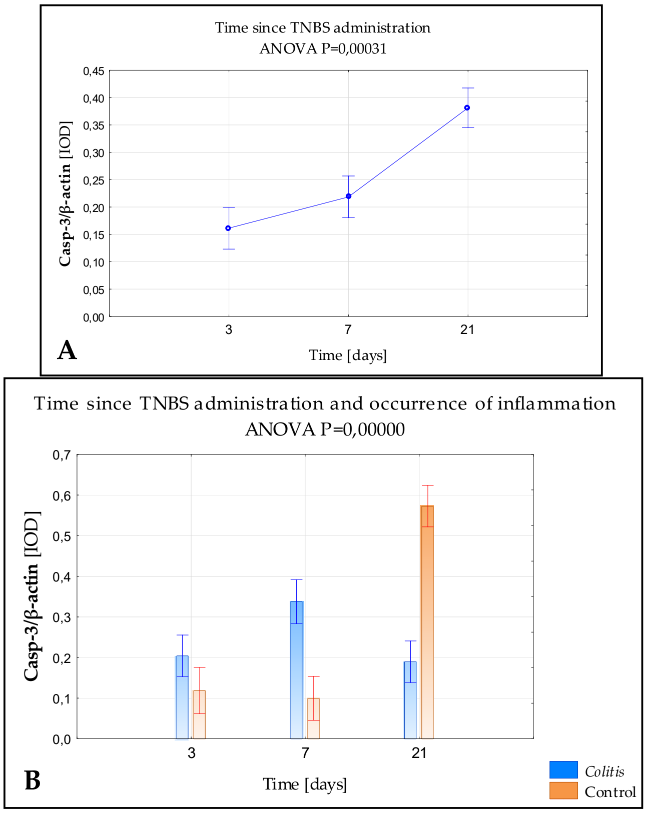

Figure S8. Results of the densitometric analysis for **Caspase-3** expression in the large intestinal wall expressed (mean ± SE) as integrated optical density (IOD). ANOVA analysis. **A** – Influence of time since TNBS/saline solution administration. **B** – Influence of interaction between the time since TNBS/saline solution administration and the occurrence of inflammation

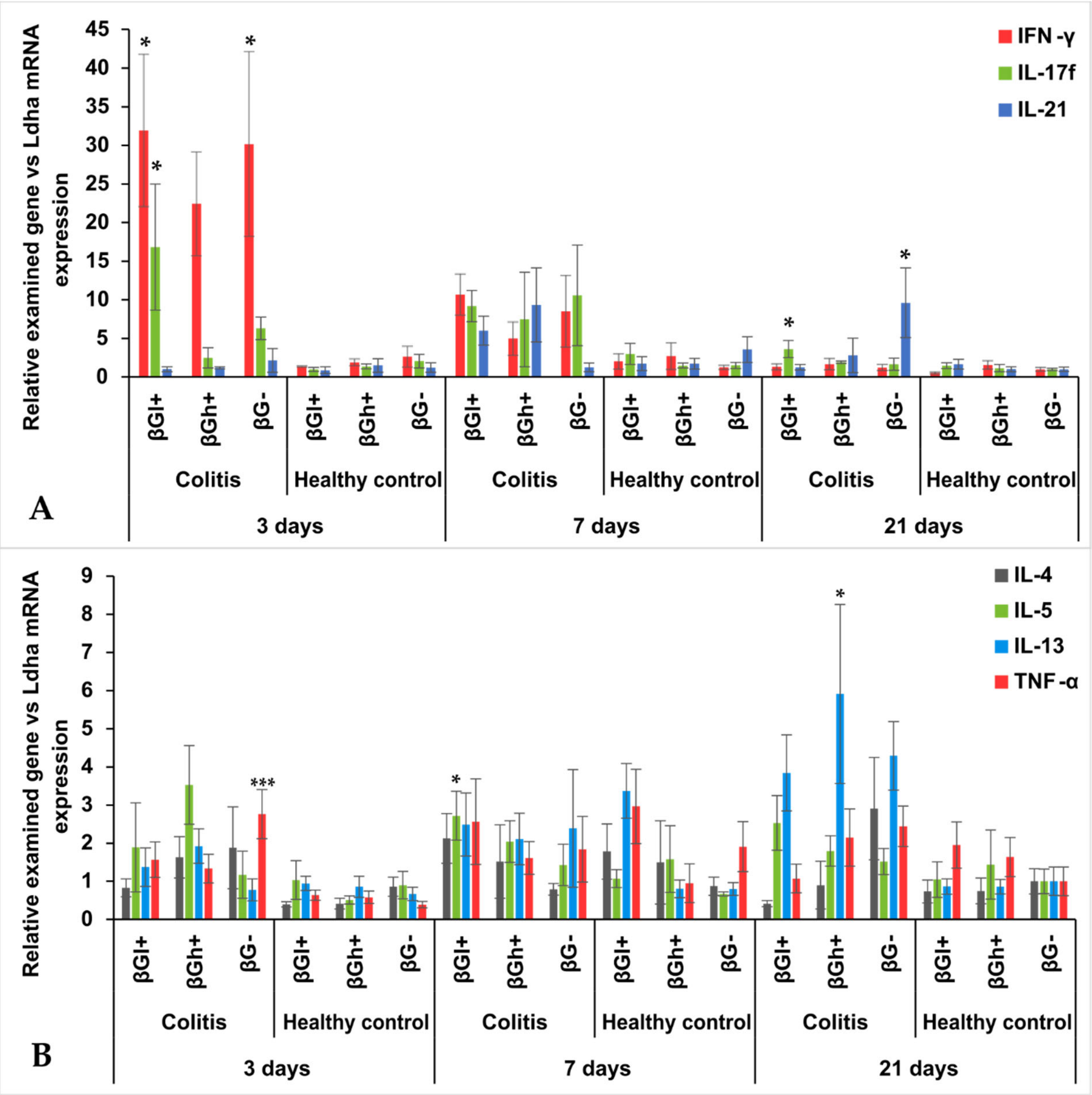

Figure S9. Relative expression of cytokine genes vs Ldha mRNA. Data presented in arbitrary unites as a ratio of target gene expression to expression of the reference gene (Ldha) with 21 days control βG- group calculated as 1 (means ± SE). **A.** *Ifng, Il17f, Il21;* **B.** *Il4, Il5, Il13, Tnf.* *Significantly different from the control group (control βG-) at the same time point according to the Dunnett post hoc test (*P<0.05, ***P<0.001).
